# Evaluation and Intercomparison of MODIS and GEOV1 Global Leaf Area Index Products over Four Sites in North China

**DOI:** 10.3390/s150306196

**Published:** 2015-03-13

**Authors:** Zhenwang Li, Huan Tang, Baohui Zhang, Guixia Yang, Xiaoping Xin

**Affiliations:** National Hulunber Grassland Ecosystem Observation and Research Station, Institute of Agricultural Resources and Regional Planning, Chinese Academy of Agricultural Sciences, Beijing 100081, China; E-Mails: lizhenwang10@hotmail.com (Z.L.); tanghuan_2011@hotmail.com (H.T.); zhangbaohui@caas.cn (B.Z.); yangguixia@caas.cn (G.Y.)

**Keywords:** LAI, MODIS, GEOV1, evaluation, intercomparison, Validation network of Remote sensing Products in China (VRPC)

## Abstract

This study investigated the performances of the Moderate Resolution Imaging Spectroradiometer (MODIS) and GEOLAND2 Version 1 (GEOV1) Leaf Area Index (LAI) products using ground measurements and LAI reference maps over four sites in North China for 2011–2013. The Terra + Aqua MODIS and Terra MODIS LAI retrieved by the main algorithm and GEOV1 LAI within the valid range were evaluated and intercompared using LAI reference maps to assess their uncertainty and seasonal variability The results showed that GEOV1 LAI is the most similar product with the LAI reference maps (R^2^ = 0.78 and RMSE = 0.59). The MODIS products performed well for biomes with low LAI values, but considerable uncertainty arose when the LAI was larger than 3. Terra + Aqua MODIS (R^2^ = 0.72 and RMSE = 0.68) was slightly more accurate than Terra MODIS (R^2^ = 0.57 and RMSE = 0.90) for producing slightly more successful observations. Both MODIS and GEOV1 products effectively followed the seasonal trajectory of the reference maps, and GEOV1 exhibited a smoother seasonal trajectory than MODIS. MODIS anomalies mainly occurred during summer and likely occurred because of surface reflectance uncertainty, shorter temporal resolutions and inconsistency between simulated and MODIS surface reflectances. This study suggests that further improvements of the MODIS LAI products should focus on finer algorithm inputs and improved seasonal variation modeling of MODIS observations. Future field work considering finer biome maps and better generation of LAI reference maps is still needed.

## 1. Introduction

The leaf area index (LAI), which is defined as the one-sided green leaf area per unit ground area [1,2], is a crucial parameter for describing vegetation structures. The index is closely associated with vegetative photosynthesis, transpiration, and the land surface energy balance [3,4], and is important in modeling biophysical processes and earth system productivity [5,6]. Satellite remote sensing is one of the most effective methods of monitoring terrestrial seasonal and inter-annual variability within regional to global domains [7]. Several global LAI products have been produced from remote sensing data obtained from different sensors, including SPOT/VEGETATION [8,9] and TERRA-AQUA/MODIS [10]. The MODIS LAI products were reprocessed for Collection 5 (C5) and released in 2007 based on previous validation reports and algorithm refinements. Most recently, the GEOLAND2 Version 1 (GEOV1) LAI product derived from SPOT/VEGETATION sensors was released to capitalize on the strong performances of several existing LAI products while limiting the situations where products show deficiencies [9]. However, because of the varying definitions of LAI products (true LAI or effective LAI) and different data sources used to produce these products, LAI products can vary significantly. Product validation for accuracy and uncertainty is thus required so that users can identify the most appropriate product, or a combination of products, to use for their applications [11].

To assess uncertainties in the LAI products of various satellite products, direct comparisons with *in situ* measurements or reference maps are typically conducted. A series of validation projects have been created, including the BigFoot Project [12], Validation of Land European Remote Sensing Instruments (VALERI) project [13], and various individual validation activities which were summarized by Garrigues *et al.* [7] and Baret *et al.* [14]. Using these datasets, many subsequent efforts have been made to validate global and regional LAI products, and various levels of accuracy and uncertainty have been reported [15,16,17,18]. To determine the best practices and guidelines and thereby form a unified validation framework, the Land Product Validation (LPV) sub-group of the Committee for Earth Observation Satellites (CEOS) developed a validation strategy [19], and four main validation stages were defined [11]. More recently, the On Line Interactive Validation Exercise (OLIVE) platform was established dedicated to compiling existing validation datasets and validating global biophysical products, such as the LAI and Fraction of Absorbed Photosynthetically Active Radiation (FAPAR), following the guidelines established by the CEOS-LPV community [20].

Continuous LAI product validation is still needed to improve the algorithms and to provide feedback to users and developers. However, because field validation efforts for direct validation were time and resource intensive, large-scale and uniformly organized field validation activities in recent years have been sparse [17,21,22]. Additionally, regional validations that consider local surface and climatic conditions are necessary. In China, such regional validations are important for understanding the performance of such products in this region. Ultimately, product intercomparisons are also important to evaluate the temporal and spatial consistency between products and to allow land surface model users to properly combine multiple LAI products while extending the temporal and spatial scales [7,23].

The Validation network of Remote sensing Products in China (VRPC) was founded in 2012, and four remote sensing stations with representative surface and good observation facilities in heterogeneous areas were included in the network for the first period of the project execution (2012–2014). Each station covers an area of 3 km × 3 km or 2 km × 2 km, and the land cover includes oasis cropland in the northwest, cropland in the north, and cropland and grassland in the northeast [24]. Using regional multi-resolution satellite images and ground observations, LAI reference maps for each station were generated. This study evaluated and intercompared the MODIS (Terra MODIS and Terra + Aqua MODIS) and GEOV1 LAI products using high-resolution field measurements and LAI reference maps of northern China for the period 2011–2013 within different seasonal and inter-annual cycles. The accuracy of these products was assessed through comparisons with high-resolution LAI reference maps derived from field measurements. Product intercomparisons were also conducted over seasonal and inter-annual cycles.

## 2. Materials and Methods

### 2.1. Study Sites 

**Figure 1:**
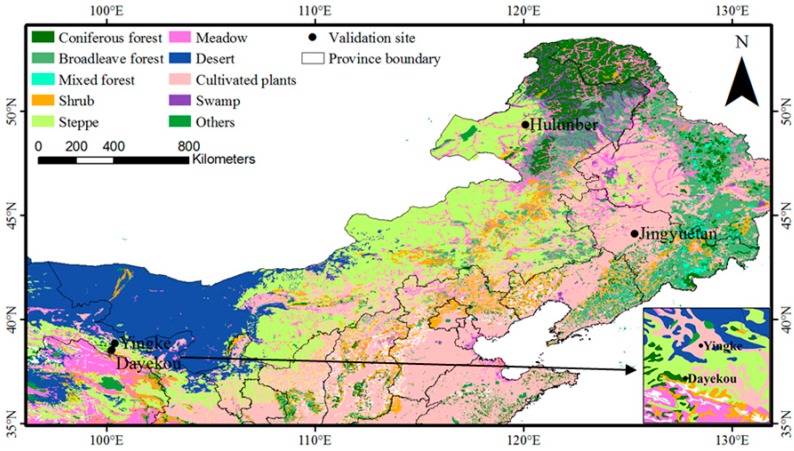
Validation site locations (vegetation type was obtained from a digitized 1:1,000,000 vegetation map of the People’s Republic of China [26]).

The following four sites were examined in this study: Yingke (oasis farmland, corn), Hulunber (meadow steppe), Jingyuetan (farmland, corn), and Dayekou (coniferous forest, *Picea crassifolia*). The first three sites were selected from the VRPC, and Ma *et al.* [24] provided detailed descriptions of these sites. The Dayekou site was selected from Heihe Watershed Allied Telemetry Experimental Research (HiWATER) [25] remote sensing station data. The site is positioned 35 km from the Yingke site and is considered a core VRPC observation field for the upcoming execution period. The four sites are located throughout northern China, covering sub-humid (Hulunber and Jingyuetan) and semi-arid (Yingke and Dayekou) climate zones. All of the sites are characterized by flat topography and vegetation canopy. The locations and characteristics of these four sites are shown in Figure 1 and Table 1.

**Table 1:** Validation site characteristics.

Site	Latitude	Longitude	Elevation(m)	Land Cover	Site Size
Dayekou	38.5337°N	100.2502°E	2835.2	Coniferous Forest	2 km × 2 km
Yingke	38.8571°N	100.4103°E	1519.1	Cropland (corn)	2 km × 2 km
Hulunber	49.3533°N	120.1246°E	650	Meadow steppe	3 km × 3 km
Jingyuetan	44.1172°N	125.3615°E	190	Cropland (corn)	3 km × 3 km

### 2.2. Field Measurements and LAI Reference Maps

In the VRPC, an LAI product validation standard and guideline named “Specifications for validation of leaf area index remote sensing products” has been formulated. This standard and guideline directs site construction and collaborative observations of the core sites and other network sites [24]. A field measurement method similar to the two-scale sampling strategy designed through the VALERI project [27] was used to collect the ground LAI and spectral data for the four sites. At each site, elementary sampling units (ESU) of 30 m × 30 m were selected based on the spatial distribution characteristics of the underlying surfaces. In each ESU, the LAI was measured at five subplots of 1 m × 1 m using the indirect optical method (LAI-2000 (Li-Cor, Lincoln, NE, USA) in Hulunber and Yingke, HemiView (Delta-T Devices Ltd, Cambridge, UK) in Dayekou or destructive harvesting method in Jingyuetan. One above-canopy and six below-canopy measurements were recorded at each point to obtain one local effective LAI value for the LAI-2000 measurements. One hemispherical photograph was obtained at each point for HemiView measurements with a 180° field-of-view fish-eye converter attached to a digital camera and then processed to derive an effective LAI estimate in the HemiView analysis software. Three to five average individual plants were collected for the destructive method. Five local LAI values were then averaged to calculate a mean value for each ESU. In addition, GPS locations (accurate to 2 m) for each ESU were recorded at the center point. Measurements were conducted in 2012 and 2013. To generate the true LAI maps, the effective LAI values (*L_e_*) measured by optical instruments at the three sites were converted to true LAIs (*LAI*) using equation 1 to correct for the presence of clumping and woody material. In Jingyuetan, field LAIs were collected using the destructive method, the clumping effect was not considered:

(1) $$LAI=(1-\alpha )L_{e}\gamma_{e}/\Omega_{e}$$

where α is the woody-to-total area ratio, γ*_e_* is the needle-to-shoot area ratio, and Ω*_e_* is the element clumping index. For grassland and cropland, we set α = 0 and γ*_e_* = 1 based on the coefficients used in the MODIS LAI algorithm, and an empirical clumping index of 0.9 was used [28]. For coniferous forest, the effects of foliage clumping and woody elements were corrected based on the information extracted from photographs using HemiView analysis software.

Generally, the following three transfer methods are used to generate high-resolution LAI maps from ground measurements and high-resolution satellite imagery: empirical methods, physical models, and hybrid approaches [16,29]. Empirical models are generally developed using correlations between remote-sensing-based vegetation indices (VI) and LAI ground measurements. For physical models, models based on the geometrical optics (GO) model or radiative transfer (RT) model are typically used, and a relationship between LAI and surface reflectance was determined by solving the GO or RT equation. Ground data are mainly used to calibrate model parameters [30,31]. A hybrid approach of physical and empirical methods has also been developed to generate high-resolution LAI maps [32,33].

In this study, HJ-1A/1B CCD and Landsat8 OLI satellite images with a 30 m spatial resolution were obtained to generate LAI reference maps (Table 2). At the Dayekou and Yingke sites, six HJ-1A/1B CCD images were collected from May to October of 2012. After preprocessing through geometric correction, radiometric calibration and atmospheric correction, a canopy BRDF (bidirectional reflectance distribution function) model [34] was established to formulate canopy reflectance as a function of LAI, wavelength, soil and leaf type, clumping index and sun-view geometry (Table 2). Finally, look-up tables (LUT) based on vegetation types were used to retrieve LAI measurements. Three bands of HJ-1A/1B CCD (NIR, red and green) and a land cover-map were used as the input, and the least-squares method was chosen as the cost function to obtain the LAI from the LUT. Before executing the BRDF model, prior information was used to parameterize the solution: vegetation was divided into 17 types based on the 30 m spatial resolution land-cover data included in the ChinaCover dataset, which was jointly mapped by the Chinese Academy of Sciences and Ministry of Environmental Protection of the People’s Republic of China [35]; single-leaf hemispherical albedos of each vegetation type were acquired from the random field spectral measurement; hemispherical albedos of soil were determined through a large amount of field spectral measurements and relevant studies in Heihe River Basin; clumping index values of every pixel for each vegetation growth condition were calculated from field measurements and the clumping index map over China’s landmass; G-function values were set to 1 for grasses and swamps, 0.8 for forests and 0.6 for farmlands depends on the leaf angle distribution of each vegetation type; and woody elements were eliminated based on previous studies by Chen *et al.* [36] and Breda *et al.* [37]. The details of the algorithm are presented in [34,38]. For the Hulunber site, seven HJ-1A/1B CCD satellite images were collected from June to August of 2013. Following radiometric calibration and atmospheric and geometric corrections, a regression model based on the LAI-NDVI (Normalized Difference Vegetation Index) relationship was built to generate LAI reference maps. The relationships were constructed using 120 pairs of ground-measured LAI and vegetation indices, and then the retrieved LAIs were improved with another 80 field-measured LAI values using a linear regression equation for every scene. Finally, the improved LAI reference maps were validated using the remaining 42 pairs of LAI validation data [39]. For the Jingyuetan site, an empirical regression approach was also employed using 79 field-measured LAIs and two Landsat8 OLI images to retrieve the LAI maps. An enhanced vegetation index (EVI) was also used [40]. 

**Table 2:** Characteristics of the LAI reference maps of the validation sites (In the BRDF equation, ρ represents the canopy reflectance; G is the G-function and represents the mean projection of a unit leaf area along the viewing or the illuminating directions; $\lambda_{0}$ represents the clumping index; and $\rho_{g}$ and $\rho_{v}$ represent the hemispherical albedos of soil background and leaf, respectively).

Site	High-Resolution Image Sensors	Date	Algorithm	Clumping Index	Reference
Dayekou and Yingke	30 m HJ-1 A/B CCD	2012/5/20; 2012/6/29; 2012/7/19; 2012/8/29; 2012/9/30; 2012/10/15	Canopy BRDF model $\rho =f(LAI,G,\lambda_{0},\rho_{g},\rho_{v})$	0.5–0.97	[37,38]
Hulunber	30 m HJ-1 A/B CCD	2013/6/4; 2013/6/23; 2013/7/13; 2013/7/21; 2013/8/11; 2013/8/19	Regression approach LAI=5.37×ln(NDVI)+3.71	0.9	[39]
Jingyuetan	30 m Landsat8 OLI	2013/6/17; 2013/7/12	Regression approach $LAI=8.32\times EVI^{2.08}$	NA	[40]

### 2.3. MODIS LAI Products

The MODIS LAI product suite for 2011–2013 with Terra C5 (MOD15 C5) and Terra + Aqua C5 (MCD15 C5) data, which were collected from the USGS Land Processes Distributed Active Archive Center (LP DAAC) [41] was used. The products have a resolution of 1 km and a temporal interval of eight days. Two algorithms were used to generate the products: the main algorithm and backup algorithm. The main algorithm is based on LUT data simulated through a three-dimensional radiative transfer model [42]. The MODIS LAI C5 algorithm refined the radiative transfer (RT) simulations in order to improve the consistency between MODIS and simulated surface reflectances, and the LUT for all biomes were recalculated with a new stochastic RT model to better depict three-dimensional effects [43]. Red and NIR atmospherically corrected MODIS reflectance and corresponding illumination-view geometry data were used as LUT inputs. Vegetation clumping was accounted for at the shoot and canopy scales for each biome type. When the main algorithm fails, a backup algorithm is triggered to estimate the LAI based on the same radiative transfer model simulated LAI-NDVI relationships. In this study, only the main algorithm retrievals were considered (quality control (QC) value < 64).

### 2.4. GEOLAND2 Version1 (GEOV1) LAI Product

The GEOV1 LAI product, which is distributed by the Copernicus Global Land portal [44], is derived from SPOT/VEGETATION sensor data at 10-day intervals and a 1-km spatial resolution. GEOV1 products are estimated using a neural network of values issued through CYCLOPES V3.1 and MODIS C5 products to take advantage of the unique strengths of each product while limiting their deficiencies [9,20]. BELMANIP2 sites that represent the possible range of surface types and conditions were used to generate fused products [14], which complement the temporal continuity of the previous VEGETATION CYCLOPES product (1999–2008) for the 1998–2014 period. Algorithm inputs correspond to the directionally normalized top of canopy reflectance in the red, NIR and SWIR bands and solar zenith angles. Preprocessing tasks include cloud screening, atmospheric correction, and directional normalization. GEOV1 LAI products are formed from a combination of lower LAI value CYCLOPES, among which leaf clumping is generally marginal. For larger MODIS LAI values, among which leaf clumping may be more significant (particularly in forest canopies), the GEOV1 LAI product generates values closer to the true LAI [45]. Similarly, retrievals within the valid range (LAI < 7.0) are considered in this study.

### 2.5. Products Validation and Intercomparison

Because of the different projection systems and data processing software used by the MODIS and GEOV1 LAI products, the images obtained for the two products within the region of interest may include pixel mismatching. In this study, we used an area-weighted average method [46] (Equation (2)) to extract pixel values of each site for each product, the geolocation of MODIS pixel was set as the target pixel (reference), and GEOV1 pixels were set as the source pixels (to be weighted):

(2) $$V=\frac{\sum_{i=1}^{n}A_{i}v_{i}}{\sum_{i=1}^{n}A_{i}}$$

where *V* represents the area-weighted pixel value, *v_i_* represents the LAI value retrieved from source pixel *i*, *A_i_* refers to overlaid area of the source pixel *i*, and *n* refers to the number of pixels that are included in the target pixel.

After extracting the LAI values of each site for each product, the product LAI values were compared with LAI reference maps to assess their uncertainty and consistency. First, valid LAI values for the validation site closest to the date of the ground measurements were extracted and averaged, and these values were then compared to the LAI values averaged from the reference maps to quantitatively evaluate the product uncertainty. Second, all valid LAI product values for the sites were compared, and the level of bias among them was calculated. Finally, temporal product profiles were determined to evaluate continuity and seasonal variations. Two statistical indicators were used to quantitatively evaluate the uncertainty: the mean absolute error (MAE) and root mean square error (RMSE) (Equations (3) and (4)):

(3) $$RMSE=\sqrt{\frac{\sum_{i=1}^{n}{\left(LAI_{s1,i}-LAI_{obs(s2),i}\right)}^{2}}{n}}$$

(4) $$MAE=\frac{\sum_{i=1}^{n}|LAI_{s1,i}-LAI_{obs(s2),i}|}{n}$$

where $LAI_{s1,i}$ is the LAI value retrieved from product 1 on date i, and $LAI_{obs(s2),i}$ refers to the field-observed LAI or the LAI value retrieved from product 2 on date i.

## 3. Results and Analysis

### 3.1. Characteristics of the LAI Reference Maps

**Figure 2:**
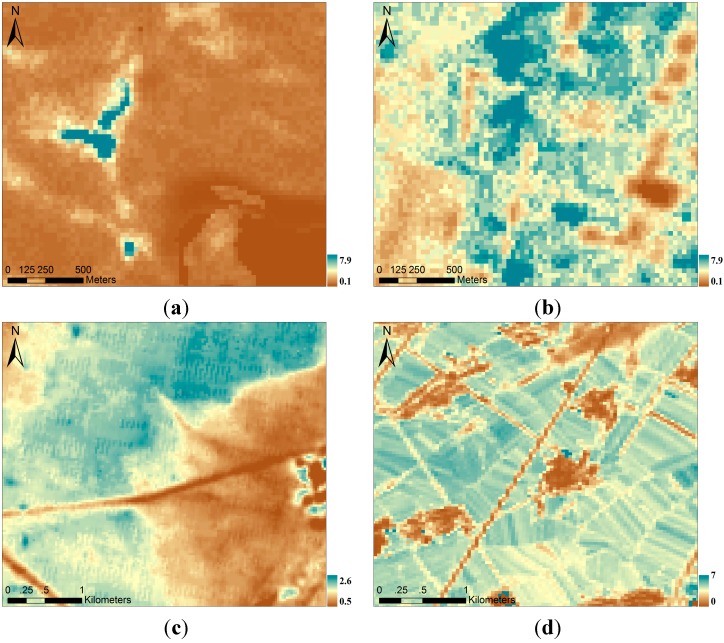
LAI reference maps of each site: (**a**) June 2012, Dayekou; (**b**) June 2012, Yingke; (**c**) July 2013, Hulunber; and (**d**) July 2013, Jingyuetan.

A total of 14 LAI reference maps (Table 3) of the four validation sites were collected from 2012 to 2013, one representative map of each site is shown in Figure 2. During the growing season, the true LAI values ranged from 0.9 to 2.1 for coniferous forests, 0.1–4.6 for croplands, and 1.0–2.2 for meadow steppes (Table 3). The highest LAI values were recorded from mid-June to mid-August, and the start of the vegetation growing season revealed a rapidly increasing LAI. A rapid LAI decline also occurred at the end of the vegetation growing season. Using field measurements, the accuracy of the LAI reference maps was estimated. For the Dayekou and Yingke sites, each LAI reference map was generated from the same HJ-1 A/B image. Reference map validation data derived from field measurements show that mean absolute error (MAE) values for forest and farmland are 0.44 and 0.56, respectively, with relatively low standard deviations of 0.29 and 0.53, respectively, for the absolute error [38]. For the Hulunber site, following retrieval and improvement tasks using ground measurements, the LAI reference maps agreed well with the field-measured LAIs (MAE value of 0.29 and MAR standard deviation of 0.20). The LAI reference maps for the Jingyuetan site are also highly accurate. The MAE between the field-measured and retrieved LAI was 0.49, with a standard deviation of 0.65.

**Table 3:** Characteristics of the LAI reference maps for the validation sites (“STDEV” under “Accuracy (STDEV)” refers to the standard deviation).

Site	Date	Mean LAI	Accuracy (MAE)	Accuracy (STDEV)
Dayekou	2012/5/20; 2012/6/29; 2012/7/19; 2012/8/29; 2012/9/30; 2012/10/15	1.0; 1.2; 2.1; 1.8; 2.0; 0.9	0.44	0.29
Yingke	2012/5/20; 2012/6/29; 2012/7/19; 2012/8/29; 2012/9/30; 2012/10/15	0.1; 4.2; 4.6; 3.1; 1.6; 0.2	0.56	0.53
Hulunber	2013/6/4; 2013/6/23; 2013/7/13; 2013/7/21; 2013/8/11; 2013/8/19	1.0; 1.7; 2.1; 2.0; 2.2; 2.2	0.29	0.20
Jingyuetan	2013/6/17; 2013/7/12	0.9; 4.3	0.49	0.65

### 3.2. Direct Comparisons

**Figure 3:**
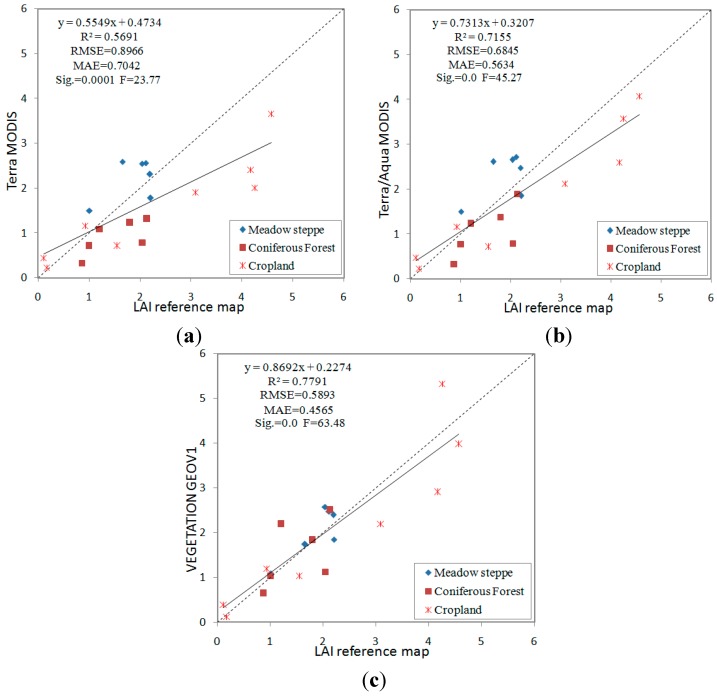
Evaluation of the LAI products using LAI reference maps: (**a**) Terra MODIS; (**b**) Terra + Aqua MODIS; and (**c**) VEGETATION GEOV1.

To assess the overall quality of the MODIS and GEOV1 LAI products, we conducted direct comparisons between the products using LAI reference maps. After checking the quality control layers for the two products, all pixels for corresponding product scenes of the validation sites were found to have the highest quality (QC = 0). The comparison results show that all of the product results agreed reasonably well with the LAI reference maps (Figure 3). The lowest degree of uncertainty compared with the LAI reference maps was achieved by the GEOV1 product (RMSE = 0.59, MAE = 0.46), followed by the Terra + Aqua MODIS (RMSE = 0.68, MAE = 0.56) and Terra MODIS products (RMSE = 0.90, MAE = 0.70). However, a slight underestimation was generated by the MODIS product, and this discrepancy increased when using a larger LAI. Figure 3a shows that uncertainty with Terra MODIS mainly occurred with LAI values greater than 3. This finding may be due to the high MODIS retrieval sensitivity to surface reflectance precision for large LAI ranges, as minor variations in the surface reflectance generate LAI variations in the saturation domain [7,47]. GEOV1 appears to be the most accurate product, especially the obvious improvement in the high LAI domain compared to MODIS. The neural network (NNT) algorithm, whose training dataset is derived from fused CYCLOPES and MODIS products with varied weights [9] and an additional SWIR band (to account for background reflectance) [48], may be the key to the favorable performance of GEOV1.

In addition to the overall performance results, product uncertainty by vegetation class is shown in Table 4. Because the Jingyuetan and Yingke sites are composed of homogeneous corn, the two sites were merged as a cropland biome. GEOV1 and MODIS estimate meadow steppe and coniferous forest more accurately than cropland, as the former two biomes generate low LAI values. In terms of accuracy, the products are ranked in the following manner: GEOV1 > Terra + Aqua MODIS > Terra MODIS. GEOV1 estimates cropland with the highest accuracy, followed by Terra + Aqua MODIS. Terra MODIS performs the worst in this area, producing an RMSE value greater than one, and the uncertainty was primarily produced as a result of largely arose from LAI values greater than 3. Moreover, the MODIS product results varied with respect to the meadow steppe and cropland, which were classified as one biome in the MODIS LAI/FPAR algorithm. MODIS overestimated meadow steppe and underestimated cropland during the rapid growth period. Similar observations were reported in Yang *et al.* [21] for sites with corn, winter wheat, and grass, which may have been caused by the effect of discrepant canopy structure between grassland (random distribution with minimal stem-trunk-branch area fraction) and cropland during the mid-growth stage (compact random distribution with substantial leaf-stem fraction) on the MODIS LAI retrieval. Because of the small number of points and limited grassland and cropland types available, additional observations in this area are required.

**Table 4:** MODIS and GEOV1 comparisons with LAI reference maps.

	n	Terra + Aqua/MODIS		Terra/MODIS		VEGETATION/GEOV1	
		MAE	RMSE	MAE	RMSE	MAE	RMSE
Meadow steppe	6	0.5497	0.5910	0.4858	0.5409	0.2705	0.3182
Coniferous forest	6	0.4607	0.6070	0.6025	0.7058	0.4301	0.5832
Cropland	8	0.6506	0.7957	0.9444	1.1901	0.6158	0.7328

### 3.3. Intercomparison of MODIS and GEOV1 LAI

**Figure 4:**
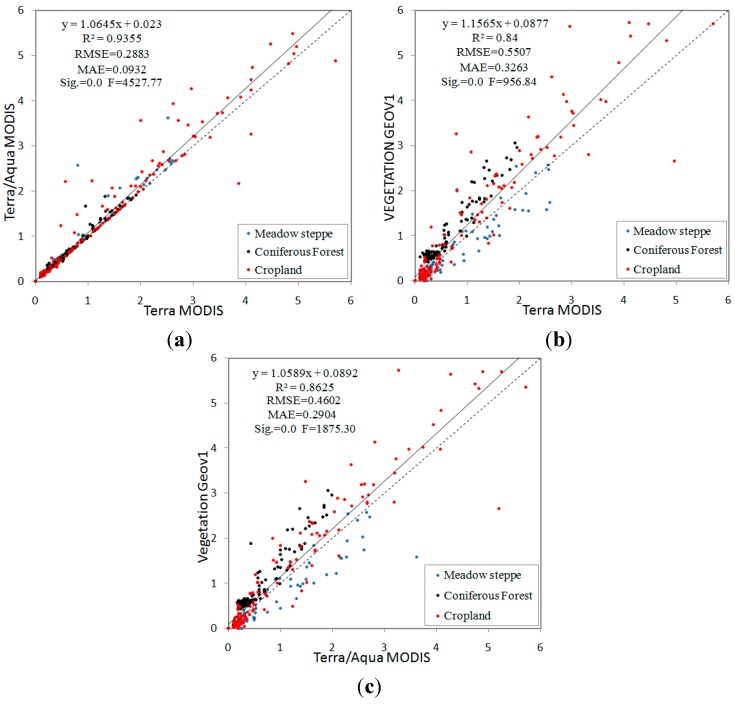
Intercomparison of LAI products: (**a**) Terra MODIS *vs.* Terra + Aqua MODIS; (**b**) Terra MODIS *vs.* VEGETATION GEOV1; and (**c**) Terra + Aqua MODIS *vs.* VEGETATION GEOV1.

To assess discrepancies between the MODIS and GEOV1 products, scatterplots for the products were generated (Figure 4). Overall, no significant discrepancies existed between the Terra and Terra + Aqua MODIS LAI product results (R^2^ = 0.94, RMSE = 0.29), although the Terra + Aqua MODIS results were slightly higher than the Terra MODIS results, reducing the underestimation of the LAI retrievals by Terra MODIS. Yang *et al.* [49] noted that the number of high-quality retrievals generated for the growing season is largely restricted by aerosol contamination, and the combined Terra-Aqua product minimizes this effect by increasing the number of high-quality retrievals by 10%–20%. Higher overall estimations were generated by the GEOV1 product than by the MODIS product, with a strong correlation R^2^ = 0.84 and 0.86 between the GEOV1 and Terra MODIS and Terra + Aqua MODIS products respectively; a relatively large scattering pattern is observed for high LAI values. This higher estimation was generally produced for coniferous forest and cropland areas, potentially due to the different canopy models [7] and input surface reflectances [50] used in each retrieval algorithm. The SWIR band used in the GEOV1 algorithm, which reduced the soil and understory background interruption, also improved the accuracy.

### 3.4. Temporal Consistency Test

**Figure 5:**
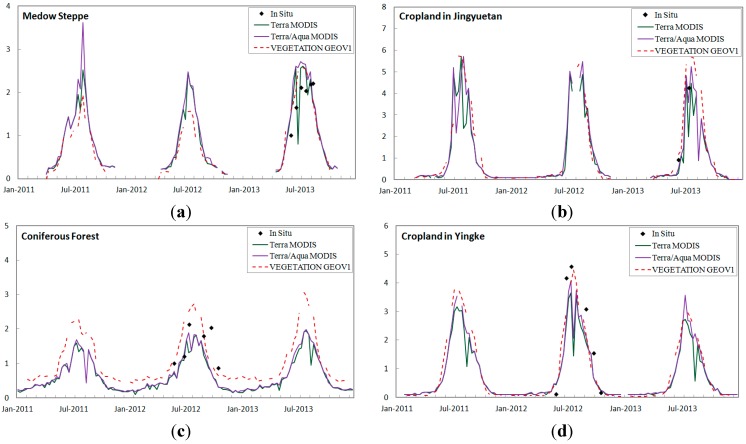
Inter-annual and seasonal variations generated by the LAI products for 2011–2013: (**a**) meadow steppe; (**b**) cropland in Jingyuetan; (**c**) conifer forest; and (**d**) cropland in Yingke.

Inter-annual and seasonal variations among the MODIS and GEOV1 LAI products for the four sites from 2011–2013 are shown in Figure 5 to determine the temporal consistency. At the four sites, both the MODIS and GEOV1 products follow the seasonal trajectories of the LAI maps reasonably well, and GEOV1 presented smoother variability. For the two MODIS products, even when only MODIS LAI values retrieved through the main algorithm were used, significant fluctuations were recorded during the peak growing season. For the GEOV1 products, NNT training could mitigate the MODIS product’s instability when a longer composite period is applied [50,51]. However, for the coniferous forest area, GEOV1 generated larger ranges than MODIS year-round. The site may be viewed as a mixture of Qinghai Spruce and bare soils due to the sparse forest cover in the area, potentially causing the MODIS product to underestimate *in situ* LAI measurements [52]. This result may also be attributable to the limited leaf clumping identified by the CYCLOPES LAI product; as a result, the GEOV1 training for low LAI values was affected [9]. For meadow steppe and crop areas, the strong agreement between the GEOV1 and MODIS results may be due to the limited leaf clumping in the two biomes [42,50].

**Figure 6:**
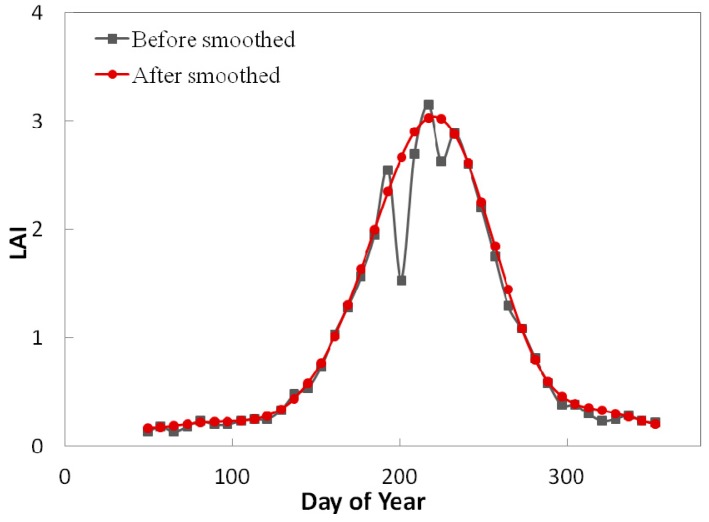
HANTS smoothing of the LAI time series data.

To determine the cause of LAI produces anomalies, we smoothed the LAI curves of the three products for each year across the four sites using the Harmonic Analysis of NDVI Time Series (HANTS) algorithm [53]. This algorithm uses a Fourier analyses to remove pronounced outliers in a time series and reconstruct a smooth curve (Figure 6). The absolute difference between the smoothed and unsmoothed LAI time series was calculated for each product and divided into five levels: no fluctuation (0–0.2), slight fluctuation (0.2–0.5), moderate fluctuation (0.5–1), large fluctuation (1–2), and intense fluctuation (2–4). The retrieval quality of the validated LAI pixels of each class were calculated by verifying the QC values as shown in Table 5. For the Terra and Terra + Aqua MODIS LAI products that were retrieved by the main algorithm, approximately 60% of the pixels had the highest quality (QC = 0 or 2), approximately 39% of the pixels were cloud contaminated (7 < QC < 32), and another 1% of the pixels were retrieved by a saturated algorithm (31 < QC < 64). For the no-fluctuation class of 0–0.2, approximately 58% of all pixels were of the highest quality and approximately 41% of the pixels were contaminated by significant or mixed cloud cover. Similarly, more than 10% of the pixels were cloud contaminated for the slight-fluctuation class of 0.2–0.5. Hence, despite the potential of cloud cover may result in poor algorithm performance, highly accurate atmospheric correction features do not generate LAI retrieval anomalies [49]. For the other classes that cause obvious LAI retrieval anomalies, numerous pixels have the highest quality (Terra MODIS, 91.64%, 58.33%, and 80% for 0.5–1, 1–2, and 2–4, respectively; and Terra + Aqua MODIS, 63.64%, 66.67%, and 25% for 0.5–1, 1–2, and 2–4, respectively), and few pixels were contaminated by clouds or saturated by the algorithm, suggesting that these factors were not a significant cause of the LAI retrieval anomalies in this study. Hence, we suggest that uncertainty in the surface reflectance, incongruities between the modeled and measured reflectance values, and shorter temporal resolutions (eight days) are the most likely causes of anomalies [47,49,50,54]. The GEOV1 product had a longer temporal compositing window, which led to an absence of cloud-contaminated pixels over the four sites, and substantial amount of pixels (65.06%) of suspect quality (QC = 4). The LAI curve demonstrated the lowest number of anomalies.

**Table 5:** Statistical pixel distribution from the LAI products curve fluctuations.

	No Fluctuation (0–0.2)	Slight Fluctuation (0.2–0.5)	Moderate Fluctuation (0.5–1)	Large Fluctuation (1–2)	Significant Fluctuation (2–4)	Total
**Terra MODIS**	374	51	12	12	5	454
Highest quality	217 (58.02%)	37 (72.55%)	11 (91.67%)	7 (58.33%)	4 (80%)	276 (60.79%)
Cloud contaminated	154 (41.18%)	14 (27.45%)	1 (8.33%)	5 (41.67%)	1 (20%)	175 (38.55%)
Algorithm saturated	3 (0.8%)	0	0	0	0	3 (0.66%)
**Terra + Aqua MODIS**	407	35	11	3	4	460
Highest quality	235 (57.74%)	31 (88.57%)	7 (63.64%)	2 (66.67%)	1 (25%)	276 (60%)
Cloud contaminated	168 (41.28%)	4 (11.43%)	3 (27.27%)	0	3 (75%)	178 (38.70%)
Algorithm saturated	4 (0.98%)	0	1 (9.09%)	1 (33.33%)	0	6 (1.30%)
**VEGETATION GEOV1**	312	34	4	2	0	352
Highest quality	107 (34.29%)	14 (41.18%)	1 (25%)	1 (50%)	0	123 (34.94%)
Suspect quality	205 (65.71%)	20 (58.82%)	3 (75%)	1 (50%)	0	229 (65.06%)

## 4. Discussion

### 4.1. LAI Reference Map Uncertainty

LAI reference map accuracy is primarily affected by ground measurement errors [7]; thus direct or indirect methods are typically used to collect ground LAI. Direct measurements through harvesting are the most accurate, and the “true LAI” can be directly used for validation [55]. Indirect allometric and optical methods are more convenient but may produce bias among the effective LAI measurements because of vegetation clumping effects, non-green elements, and optical signals saturation [56,57,58]. To determine the true LAI of a vegetation canopy, the effective LAI must be corrected for foliage clumping and non-photosynthetic elements.

For the Jingyuetan site, the “true LAI” was obtained through direct harvesting, and the mean LAI values of each sampling unit were used to generate the LAI reference maps. Because of clumping effects, negligible uncertainties may have been obtained for the Hulunber site when empirical clumping indices were used, although limited leaf clumping processes in meadow steppe areas may limit the degree of bias [50]. Understory or non-green elements may serve as the main source of uncertainty that affects the generation of LAI reference maps because the optical method accounts for stem and branch influences on light interception [59]. In cropland and grassland areas, the effect of non-green elements is negligible because only a limited number of senescent leaves and non-green tissues are present [42]. As for coniferous forests, the understory can produce substantial variations among satellite LAI products derived through the vertical integration of radiometric signals within the canopy [60,61]. 

Geolocation and spatially random errors of ground measurements and LAI reference maps will also have a profound influence on product comparisons [62,63]. In this study, ground-measured LAI values collected from each sampling unit were rigorously scrutinized and averaged to represent the field conditions. High-resolution LAI reference maps were also aggregated to the site scale.

### 4.2. Study Limitations

With the exception of major uncertainties that originate from the ground measurements and LAI reference maps described in Section 4.1, a second source of uncertainty is associated with the methods used to generate the LAI reference maps (regression and physical methods) as each method presents unique advantages and limitations [16,64]. The regression approach is straightforward and easy to compute but is site- and sensor-specific [60]. The physical method is physically based and biome-independent but potentially ill-posed [65,66]. Several studies have found that physical methods are more effective than statistical methods when studying large areas [64,67]; when studying a single site, statistical methods may be more appropriate, although the differences between the two methods must be further explored.

Uncertainty in our results may also be a result of a spatial mismatch between the LAI reference maps and moderate resolution products. Every product exhibits geometric accuracy, and a pixel geolocation error may shift pixels and potentially interrupt the analysis by introducing mixed pixels [60,68]. However, different projection systems and data processing methods applied to each product will also cause pixel mismatching. In our study, pixel shifts can affect half of all pixels, and we used an area-weighted average method to extract pixel values for each site. The large and homogeneous surface within and around the validation site can decrease the effect of geolocation errors.

This research must also be expanded to new biome types that have not yet been adequately represented, particularly woody savannas and broadleaf forest biomes with higher clumping effects and/or greater degrees of understory influence. The biomes validated in this study cannot properly represent the overall product variability, although they can serve as a reference for future validations.

### 4.3. Avenues for Future Research

Given the limitations presented in this study, future validation work must be conducted in several areas. First, more validation sites of various biome types across China must be assessed to measure the LAI product uncertainty in these regions. Second, given the variable MODIS LAI product grassland and cropland results for the mid-growth stage, field work must be conducted in more grassland and cropland areas and must be used for MODIS LAI product validation. Third, several vegetation indices that can estimate green leaf area indices [69,70] may be used in future regressions, and input data and parameters should be refined to reduce physical algorithm uncertainties. A comparison between these parameters should also be conducted to identify the best method for LAI reference map retrievals. Finally, a longer time series of validation data must be built using the algorithm developed here to further assess the interannual variations in response to climate, management, or pest outbreaks.

## 5. Conclusions 

In this study, the MODIS and GEOV1 LAI products were evaluated and intercompared using ground measurements and LAI reference maps of northern China. Generally, the GEOV1 product was the most accurate as it generated data that reflected the LAI reference maps. The MODIS products also performed well for meadow steppe, cropland and coniferous forest areas with low LAI values. However, for croplands that have LAI values larger than 3 and domains with LAI-retrieval accuracies limited by the precision of MODIS surface reflectances, Terra + Aqua MODIS produced more successful observations than Terra MODIS. The MODIS and GEOV1 products were consistent with the seasonal trajectories of the reference maps; GEOV1 generated smoother LAI variability, while the MODIS products generated more variable results during the summer. The anomalous seasonality was most likely caused by the uncertainty in the surface reflectance, incongruities between the modeled and measured reflectance values, and shorter temporal resolutions of eight days. This study recommends that further improvements of the MODIS LAI products should focus on finer algorithm inputs and improved seasonal variation modeling of MODIS observations.

## References

[1] Chen J.M., Black T.A. (1992). Defining leaf area index for non-flat leaves. Plant Cell Environ..

[2] Myneni R.B., Hoffman S., Knyazikhin Y., Privette J.L., Glassy J., Tian Y., Wang Y., Song X., Zhang Y., Smith G.R. (2002). Global products of vegetation leaf area and fraction absorbed par from year one of MODIS data. Remote Sens. Environ..

[3] Running S.W., Nemani R.R., Peterson D.L., Band L.E., Potts D.F., Pierce L.L., Spanner M.A. (1989). Mapping regional forest evapotranspiration and photosynthesis by coupling satellite data with ecosystem simulation. Ecology.

[4] Sellers P.J., Dickinson R.E., Randall D.A., Betts A.K., Hall F.G., Berry J.A., Collatz G.J., Denning A.S., Mooney H.A., Nobre C.A. (1997). Modeling the exchanges of energy, water, and carbon between continents and the atmosphere. Science.

[5] Bonan G.B. (1995). Land-atmosphere interactions for climate system models: Coupling biophysical, biogeochemical, and ecosystem dynamical processes. Remote Sens. Environ..

[6] Foley J.A., Levis S., Prentice I.C., Pollard D., Thompson S.L. (1998). Coupling dynamic models of climate and vegetation. Glob. Chang. Biol..

[7] Garrigues S., Lacaze R., Baret F., Morisette J.T., Weiss M., Nickeson J.E., Fernandes R., Plummer S., Shabanov N.V., Myneni R.B. (2008). Validation and intercomparison of global leaf area index products derived from remote sensing data. J. Geophys. Res.: Biogeosci..

[8] Baret F., Hagolle O., Geiger B., Bicheron P., Miras B., Huc M., Berthelot B., Niño F., Weiss M., Samain O. (2007). LAI, fAPAR and fCover CYCLOPES global products derived from VEGETATION part 1: Principles of the algorithm. Remote Sens. Environ..

[9] Baret F., Weiss M., Lacaze R., Camacho F., Makhmara H., Pacholcyzk P., Smets B. (2013). GEOV1: LAI and FAPAR essential climate variables and FCOVER global time series capitalizing over existing products. Part1: Principles of development and production. Remote Sens. Environ..

[10] Yang W., Huang D., Tan B., Stroeve J.C., Shabanov N.V., Knyazikhin Y., Nemani R.R., Myneni R.B. (2006). Analysis of Leaf Area Index and Fraction of PAR Absorbed by Vegetation Products From the Terra MODIS Sensor: 2000–2005. IEEE Trans. Geosci. Remote Sens..

[11] Morisette J.T., Baret F., Privette J.L., Myneni R.B., Nickeson J.E., Garrigues S., Shabanov N.V., Weiss M., Fernandes R.A., Leblanc S.G. (2006). Validation of global moderate-resolution LAI products: A framework proposed within the CEOS land product validation subgroup. IEEE Trans. Geosci. Remote Sens..

[12] Bigfoot. http://www.fsl.orst.edu/larse/bigfoot/index.html.

[13] Validation of Land European Remote Sensing Instruments. http://w3.avignon.inra.fr/valeri/.

[14] Baret F., Morissette J.T., Fernandes R.A., Champeaux J.L., Myneni R.B., Chen J., Plummer S., Weiss M., Bacour C., Garrigues S. (2006). Evaluation of the representativeness of networks of sites for the global validation and intercomparison of land biophysical products: Proposition of the CEOS-BELMANIP. IEEE Trans. Geosci. Remote Sens..

[15] Privette J.L., Myneni R.B., Knyazikhin Y., Mukelabai M., Roberts G., Tian Y., Wang Y., Leblanc S.G. (2002). Early spatial and temporal validation of MODIS LAI product in the southern Africa Kalahari. Remote Sens. Environ..

[16] Yang W., Tan B., Huang D., Rautiainen M., Shabanov N.V., Wang Y., Privette J.L., Huemmrich K.F., Fensholt R., Sandholt I. (2006). MODIS leaf area index products: From validation to algorithm improvement. IEEE Trans. Geosci. Remote Sens..

[17] Gessner U., Niklaus M., Kuenzer C., Dech S. (2013). Intercomparison of leaf area index products for a gradient of sub-humid to arid environments in west Africa. Remote Sens..

[18] Fang H., Wei S., Liang S. (2012). Validation of MODIS and CYCLOPES LAI products using global field measurement data. Remote Sens. Environ..

[19] CEOS LVP. http://lpvs.gsfc.nasa.gov/.

[20] Weiss M., Baret F., Block T., Koetz B., Burini A., Scholze B., Lecharpentier P., Brockmann C., Fernandes R., Plummer S. (2014). On Line Validation Exercise (OLIVE): A web based service for the validation of medium resolution land products. Application to FAPAR products. Remote Sens..

[21] Yang F., Yang J., Wang J., Zhu Y. (2014). Assessment and validation of MODIS and GEOV1 LAI with ground-measured data and an analysis of the effect of residential area in mixed pixel. IEEE J. Sel. Top. Appl. Earth Obs. Remote Sens..

[22] Claverie M., Vermote E.F., Weiss M., Baret F., Hagolle O., Demarez V. (2013). Validation of coarse spatial resolution LAI and FAPAR time series over cropland in southwest france. Remote Sens. Environ..

[23] Fang H., Jiang C., Li W., Wei S., Baret F., Chen J.M., Garcia-Haro J., Liang S., Liu R., Myneni R.B. (2013). Characterization and intercomparison of global moderate resolution leaf area index (LAI) products: Analysis of climatologies and theoretical uncertainties. J. Geophys. Res. Biogeosci..

[24] Ma M.G., Li X., Weizhen W., Xiao Q., Zhao K., Xin X.P. Design on validation network of remote sensing products in China. Proceedings of the 2013 8th International Symposium on Spatial Data Quality.

[25] Li X., Cheng G., Liu S., Xiao Q., Ma M., Jin R., Che T., Liu Q., Wang W., Qi Y. (2013). Heihe watershed allied telemetry experimental research (HIWETER): Scientific objectives and experimental design. Bull. Am. Meteorol. Soc..

[26] Vegetation Map of the People’s Republic of China (1:1,000,000). http://www.resdc.cn.

[27] VALERI: A Network of Sites and a Methodology for the Validation of Medium Spatial Resolution Land Satellite Products. http://w3.avignon.inra.fr/valeri/documents/VALERI-RSESubmitted.pdf.

[28] Liu R., Chen J.M., Liu J., Deng F., Sun R. (2007). Application of a new leaf area index algorithm to China’s landmass using MODIS data for carbon cycle research. J. Environ. Manag..

[29] Fang H., Liang S. (2014). Leaf area index models. Reference Module in Earth Systems and Environmental Sciences.

[30] Ganguly S., Nemani R.R., Zhang G., Hashimoto H., Milesi C., Michaelis A., Wang W., Votava P., Samanta A., Melton F. (2012). Generating global leaf area index from Landsat: Algorithm formulation and demonstration. Remote Sens. Environ..

[31] Duan S.-B., Li Z.-L., Wu H., Tang B.-H., Ma L., Zhao E., Li C. (2014). Inversion of the PROSAIL model to estimate leaf area index of maize, potato, and sunflower fields from unmanned aerial vehicle hyperspectral data. Int. J. Appl. Earth Obs. Geoinf..

[32] Qi J., Kerr Y.H., Moran M.S., Weltz M., Huete A.R., Sorooshian S., Bryant R. (2000). Leaf area index estimates using remotely sensed data and BRDF models in a semiarid region. Remote Sens. Environ..

[33] Liu Q., Liang S., Xiao Z., Fang H. (2014). Retrieval of leaf area index using temporal, spectral, and angular information from multiple satellite data. Remote Sens. Environ..

[34] Yan B., Xu X., Fan W. (2012). A unified canopy bidirectional reflectance (BRDF) model for row crops. Sci. China Earth Sci..

[35] ChinaCover. http://www.chinacover.org.cn/.

[36] Chen J.M., Govind A., Sonnentag O., Zhang Y., Barr A., Amiro B. (2006). Leaf area index measurements at fluxnet-Canada forest sites. Agric. For. Meteorol..

[37] Bréda N.J.J. (2003). Ground-based measurements of leaf area index: A review of methods, instruments and current controversies. J. Exp. Bot..

[38] Liao Y., Fan W., Xu X. Algorithm of Leaf Area Index Product for HJ-CCD Over Heihe River Basin. Proceedings of the 2013 IEEE International Geoscience and Remote Sensing Symposium (IGARSS).

[39] Li Z., Tang H., Xin X., Zhang B., Wang D. (2014). Assessment of the MODIS LAI product using ground measurement data and HJ-1A/1B imagery in the meadow steppe of Hulunber, China. Remote Sens..

[40] Ding Y., Ge Y., Hu M., Wang J., Wang J., Zheng X., Zhao K. (2014). Comparison of spatial sampling strategies for ground sampling and validation of MODIS LAI products. Int. J. Remote Sens..

[41] Land Processes Distributed Active Archive Center. https://lpdaac.usgs.gov/.

[42] Knyazikhin Y., Martonchik J.V., Myneni R.B., Diner D.J., Running S.W. (1998). Synergistic algorithm for estimating vegetation canopy leaf area index and fraction of absorbed photosynthetically active radiation from MODIS and MISR data. J. Geophys. Res. Atmos..

[43] Myneni R., Knyazikhin Y., Glassy J., Votava P., Shabanov N. (2003). User’s Guide: FPAR, LAI (ESDT: MOD15A2) 8-Day Composite NASA MODIS Land Algorithm.

[44] Copernicus Global Land Portal. http://land.copernicus.eu/global/.

[45] Camacho F., Cernicharo J., Lacaze R., Baret F., Weiss M. (2013). GEOV1: LAI, FAPAR essential climate variables and FCOVER global time series capitalizing over existing products. Part 2: Validation and intercomparison with reference products. Remote Sens. Environ..

[46] Ge Y., Avitabile V., Heuvelink G.B.M., Wang J., Herold M. (2014). Fusion of pan-tropical biomass maps using weighted averaging and regional calibration data. Int. J. Appl. Earth Obs. Geoinf..

[47] Shabanov N.V., Dong H., Wenze Y., Tan B., Knyazikhin Y., Myneni R.B., Ahl D.E., Gower S.T., Huete A.R., Aragao L.E.O.C. (2005). Analysis and optimization of the MODIS leaf area index algorithm retrievals over broadleaf forests. IEEE Trans. Geosci. Remote Sens..

[48] Baret F., Guyot G., Begue A., Maurel P., Podaire A. (1988). Complementarity of middle-infrared with visible and near-infrared reflectance for monitoring wheat canopies. Remote Sens. Environ..

[49] Yang W., Shabanov N.V., Huang D., Wang W., Dickinson R.E., Nemani R.R., Knyazikhin Y., Myneni R.B. (2006). Analysis of leaf area index products from combination of MODIS Terra and Aqua data. Remote Sens. Environ..

[50] Verger A., Baret F., Weiss M. (2008). Performances of neural networks for deriving LAI estimates from existing CYCLOPES and MODIS products. Remote Sens. Environ..

[51] Verger A., Baret F., Weiss M. (2011). A multisensor fusion approach to improve LAI time series. Remote Sens. Environ..

[52] De Kauwe M.G., Disney M.I., Quaife T., Lewis P., Williams M. (2011). , An assessment of the MODIS collection 5 leaf area index product for a region of mixed coniferous forest. Remote Sens. Environ..

[53] Menenti M., Azzali S., Verhoef W., van Swol R. (1993). Mapping agroecological zones and time lag in vegetation growth by means of fourier analysis of time series of NDVI images. Adv. Space Res..

[54] Tan B., Hu J., Huang D., Yang W., Zhang P., Shabanov N.V., Knyazikhin Y., Nemani R.R., Myneni R.B. (2005). Assessment of the broadleaf crops leaf area index product from the Terra MODIS instrument. Agric. For. Meteorol..

[55] Jonckheere I., Fleck S., Nackaerts K., Muys B., Coppin P., Weiss M., Baret F. (2004). Review of methods for *in situ* leaf area index determination: Part I. Theories, sensors and hemispherical photography. Agric. For. Meteorol..

[56] Chen J.M., Rich P.M., Gower S.T., Norman J.M., Plummer S. (1997). Leaf area index of boreal forests: Theory, techniques, and measurements. J. Geophys. Res.: Atmos..

[57] Kucharik C.J., Norman J.M., Gower S.T. (1998). Measurements of branch area and adjusting leaf area index indirect measurements. Agric. For. Meteorol..

[58] Leblanc S.G., Chen J.M. (2001). A practical scheme for correcting multiple scattering effects on optical LAI measurements. Agric. For. Meteorol..

[59] Chen J.M. (1996). Optically-based methods for measuring seasonal variation of leaf area index in boreal conifer stands. Agric. For. Meteorol..

[60] Wang Y., Woodcock C.E., Buermann W., Stenberg P., Voipio P., Smolander H., Häme T., Tian Y., Hu J., Knyazikhin Y. (2004). Evaluation of the MODIS LAI algorithm at a coniferous forest site in Finland. Remote Sens. Environ..

[61] Qi Y., Li F., Liu Z., Jin G. (2014). Impact of understorey on overstorey leaf area index estimation from optical remote sensing in five forest types in northeastern China. Agric. For. Meteorol..

[62] Tian Y., Woodcock C.E., Wang Y., Privette J.L., Shabanov N.V., Zhou L., Zhang Y., Buermann W., Dong J., Veikkanen B. (2002). Multiscale analysis and validation of the MODIS LAI product: I. Uncertainty assessment. Remote Sens. Environ..

[63] Tan B., Hu J., Zhang P., Huang D., Shabanov N., Weiss M., Knyazikhin Y., Myneni R.B. (2005). Validation of moderate resolution imaging spectroradiometer leaf area index product in croplands of alpilles, France. J. Geophys. Res. Atmos..

[64] Darvishzadeh R., Atzberger C., Skidmore A., Schlerf M. (2011). Mapping grassland leaf area index with airborne hyperspectral imagery: A comparison study of statistical approaches and inversion of radiative transfer models. ISPRS J. Photogramm. Remote Sens..

[65] Atzberger C. (2004). Object-based retrieval of biophysical canopy variables using artificial neural nets and radiative transfer models. Remote Sens. Environ..

[66] Darvishzadeh R., Skidmore A., Schlerf M., Atzberger C. (2008). Inversion of a radiative transfer model for estimating vegetation LAI and chlorophyll in a heterogeneous grassland. Remote Sens. Environ..

[67] Leonenko G., Los S.O., North P.R.J. (2013). Retrieval of leaf area index from MODIS surface reflectance by model inversion using different minimization criteria. Remote Sens. Environ..

[68] Wu H., Li Z.-L. (2009). Scale issues in remote sensing: A review on analysis, processing and modeling. Sensors.

[69] Nguy-Robertson A.L., Peng Y., Gitelson A.A., Arkebauer T.J., Pimstein A., Herrmann I., Karnieli A., Rundquist D.C., Bonfil D.J. (2014). Estimating green LAI in four crops: Potential of determining optimal spectral bands for a universal algorithm. Agric. For. Meteorol..

[70] Hagen S.C., Heilman P., Marsett R., Torbick N., Salas W., van Ravensway J., Qi J. (2012). Mapping total vegetation cover across western rangelands with moderate-resolution imaging spectroradiometer data. Rangel. Ecol. Manag..

